# Supplementary material for: Cascade electrocatalysis via AgCu single-atom alloy and Ag nanoparticles in CO2 electroreduction toward multicarbon products
Source: Nat Commun. 2023 Oct 5;14:6142. doi: 10.1038/s41467-023-41871-w (PMC10556094; doi:10.1038/s41467-023-41871-w)
Supplement: Supplementary file 1 — Supplementary information [file 41467_2023_41871_MOESM1_ESM.pdf]

Cascade electrocatalysis via AgCu single-atom alloy and Ag nanoparticles in CO_2 electroreduction toward multi-carbon products

Cheng Du,[1Δ] *Joel. P. Mills,*[1Δ] *Asfaw G. Yohannes,*[2Δ] *Wei Wei,*[1Δ] *Lei Wang,*[1] *Siyan Lu,*[1] *Jian-Xiang Lian,*[2] *Maoyu Wang,*[3] *Tao Guo,*[1] *Xiyang Wang,*[1] *Hua Zhou,*[3] *Cheng-Jun Sun,*[3] *John Z. Wen,*[1] *Brian Kendall,*[4] *Martin Couillard,*[5] *Hongsheng Guo,*[5] *ZhongChao Tan,*[1*] *Samira Siahrostami,*[2*] *Yimin A. Wu*[1,6,7*]

[1]Department of Mechanical and Mechatronics Engineering, Waterloo Institute for Nanotechnology, Materials Interfaces Foundry, University of Waterloo, Waterloo, Ontario N2L 3G1, Canada

[2]Department of Chemistry, University of Calgary, 2500 University Drive NW, Calgary, Alberta, T2N 1N4 Canada

[3]X-Ray Science Division, Argonne National Laboratory, Lemont, IL 60439, USA

[4]Department of Earth and Environmental Sciences, University of Waterloo, Waterloo, Ontario N2L 3G1, Canada

[5]Energy, Mining and Environment Research Center, National Research Council Canada, 1200 Montreal Road, Ottawa, Ontario K1A 0R6, Canada

[6]Interdisciplinary Center on Climate Change, University of Waterloo, Waterloo, Ontario N2L 3G1, Canada

[7]Department of Chemistry, University of Waterloo, Ontario N2L 3G1, Canada

[Δ] Equal contribution

* Corresponding emails: zhongchao.tan@uwaterloo.ca; samira.siahrostami@ucalgary.ca; yimin.wu@uwaterloo.ca

This Supplementary Information file includes:

Figure S1 to S17, Table S1 to S3, and Supplementary References.

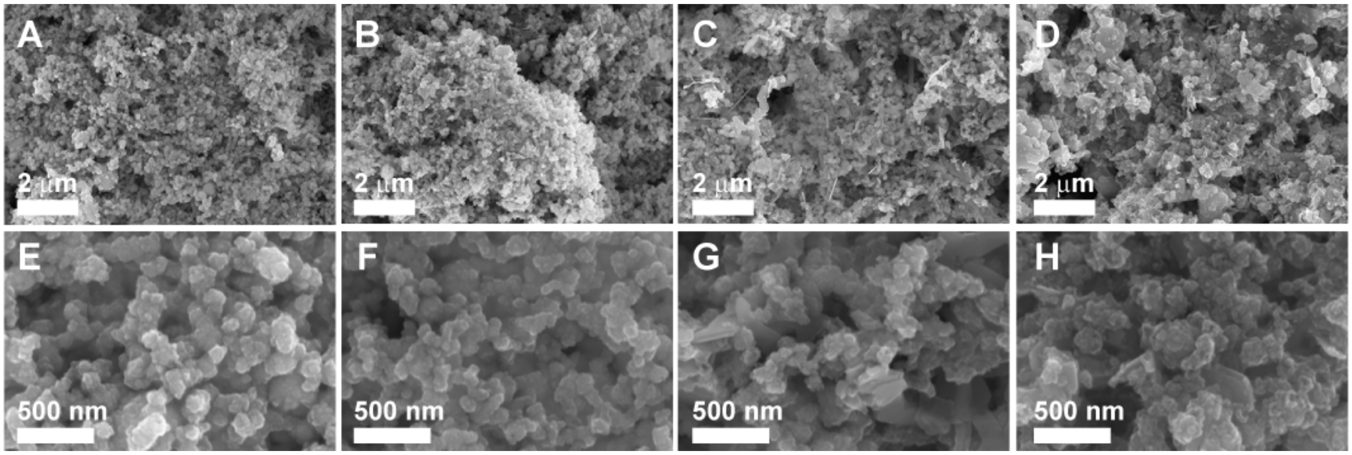

Figure S1. SEM images of Cu NP (A and E), AgCu SAA (B and F), AgCu SANP (C and G), and Ag NP (D and H).

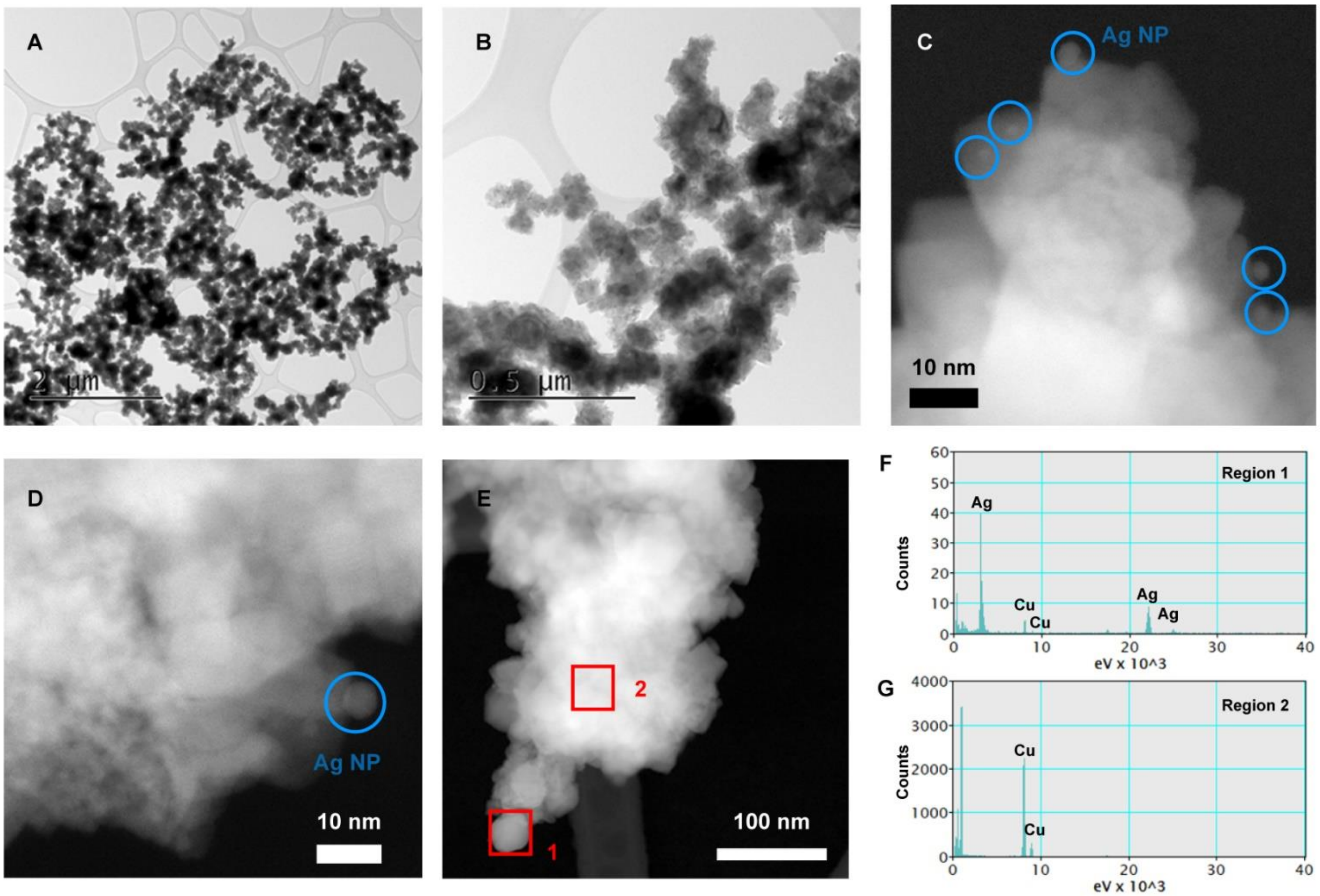

Figure S2. (A-B) BF-STEM images of AgCu SANP; (C-D) HAADF-STEM images of AgCu SANP, the representative Ag NP are marked by the circle; (E) HAADF-STEM image of AgCu SANP, region 1 represents the Ag NP, region 2 represents AgCu SAA; (F) EDX of region 1, which shows the apparent signal of Ag and Cu; (G) EDX of region 2, which only shows the signal of Cu without Ag due to the low content of Ag in AgCu SAA composition.

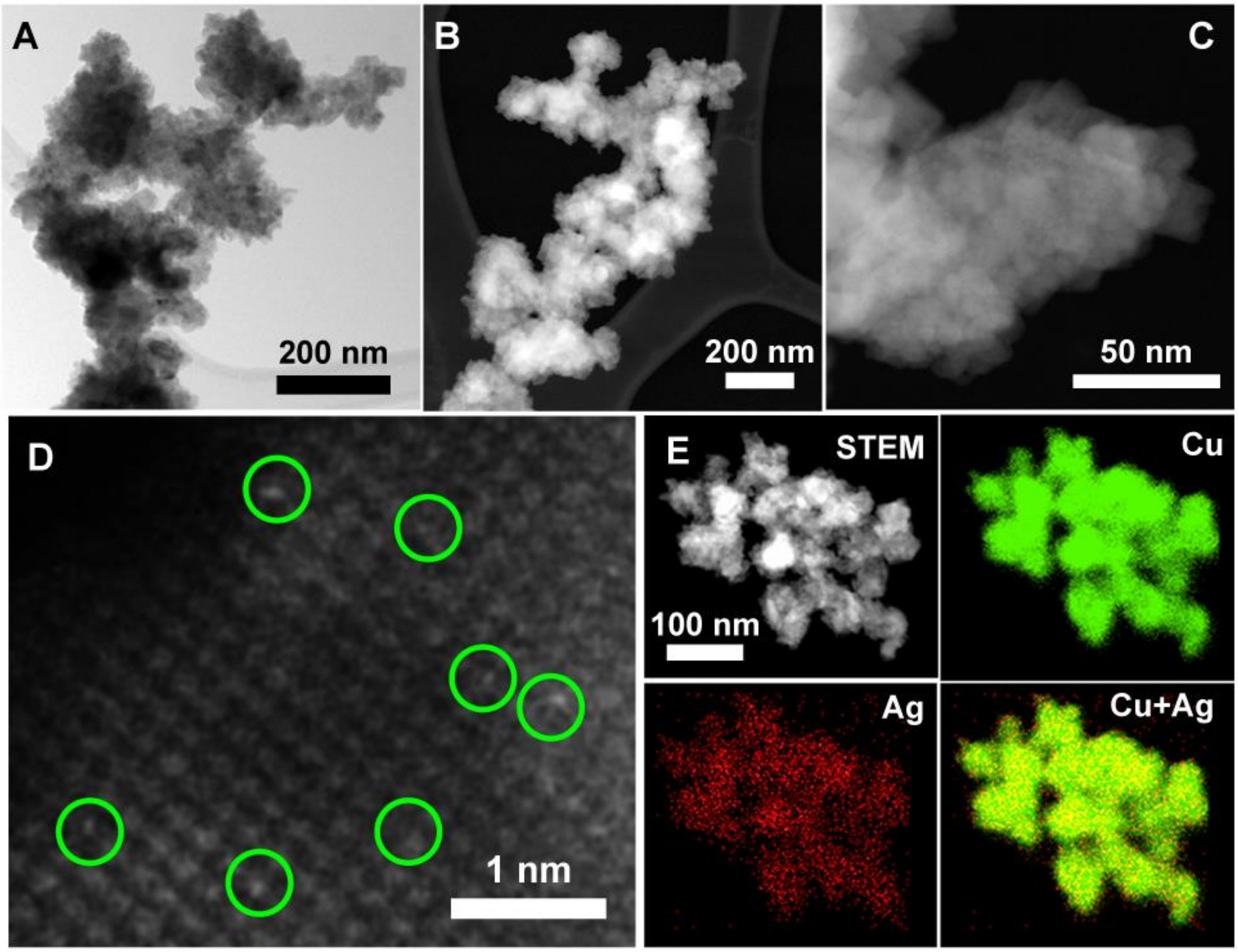

Figure S3. (A) BF-STEM image of AgCu SAA; (B-C) HAADF-STEM images with different magnifications of AgCu SAA; (D) AC-HAADF-STEM image of AgCu SAA, the bright dot marked by the circle represents the Ag single-atoms; (E) STEM and EDS element mapping images of AgCu SAA.

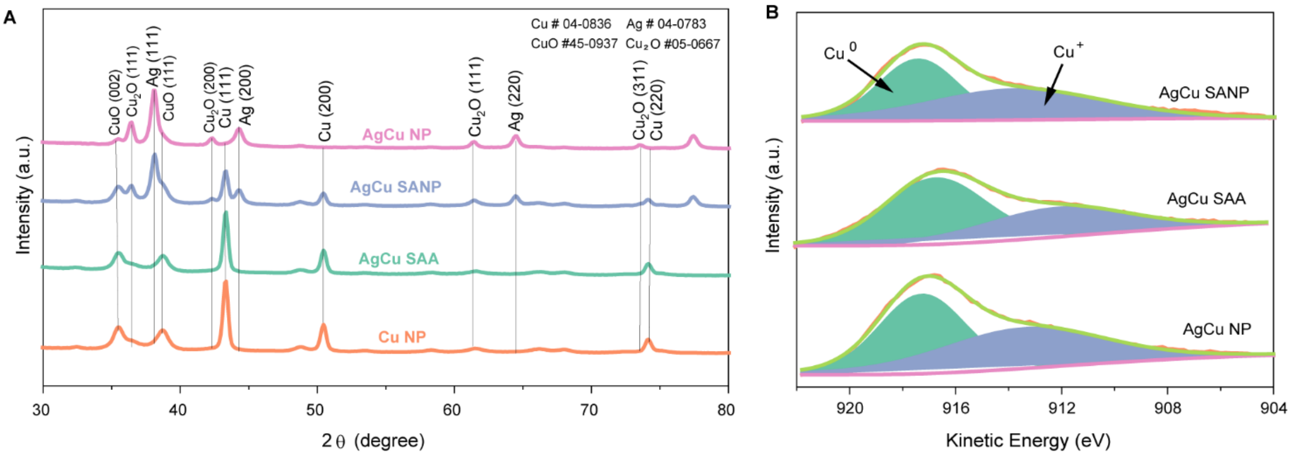

Figure S4. (A) XRD spectra of Cu NP, AgCu SAA, AgCu SANP and AgCu NP; (B) Auger spectra of AgCu SAA, AgCu SANP and AgCu NP. Source data are provided with this paper.

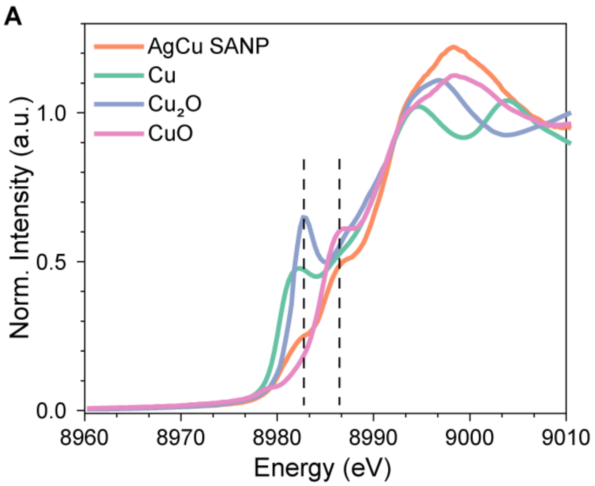

Figure S5. (A) Cu K-edge XANES spectra of AgCu SANP, Cu foil, Cu_2O and CuO; (B) Cu K-edge EXAFS experimental and fitting spectrum of AgCu SANP. Source data are provided with this paper.

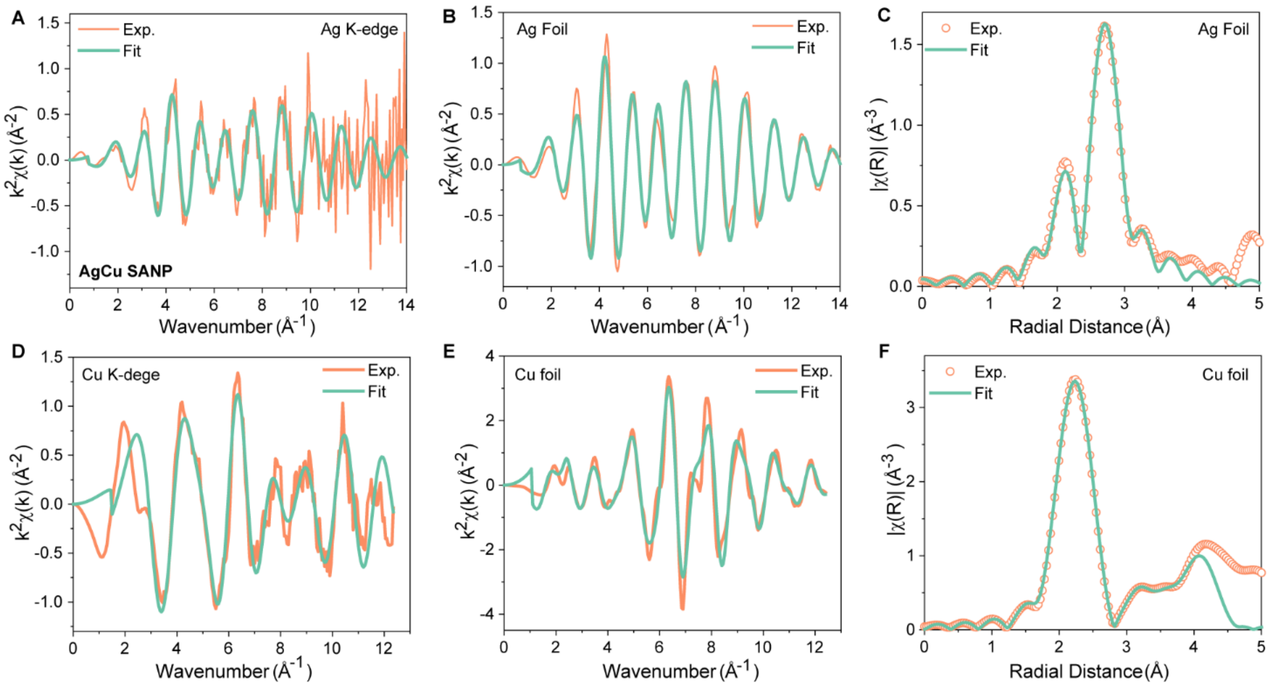

Figure S6. (A-B) Experimental and fitting spectra of Ag K-edge in k space of AgCu SANP (A) and Ag foil (B); (C) Experimental and fitting spectra of Ag K-edge in r space of Ag foil; (D-E) Experimental and fitting spectra of Cu K-edge in k space of AgCu SANP (D) and Cu foil (E); (F) Experimental and fitting spectra of Cu K-edge in r space of Cu foil. Source data are provided with this paper.

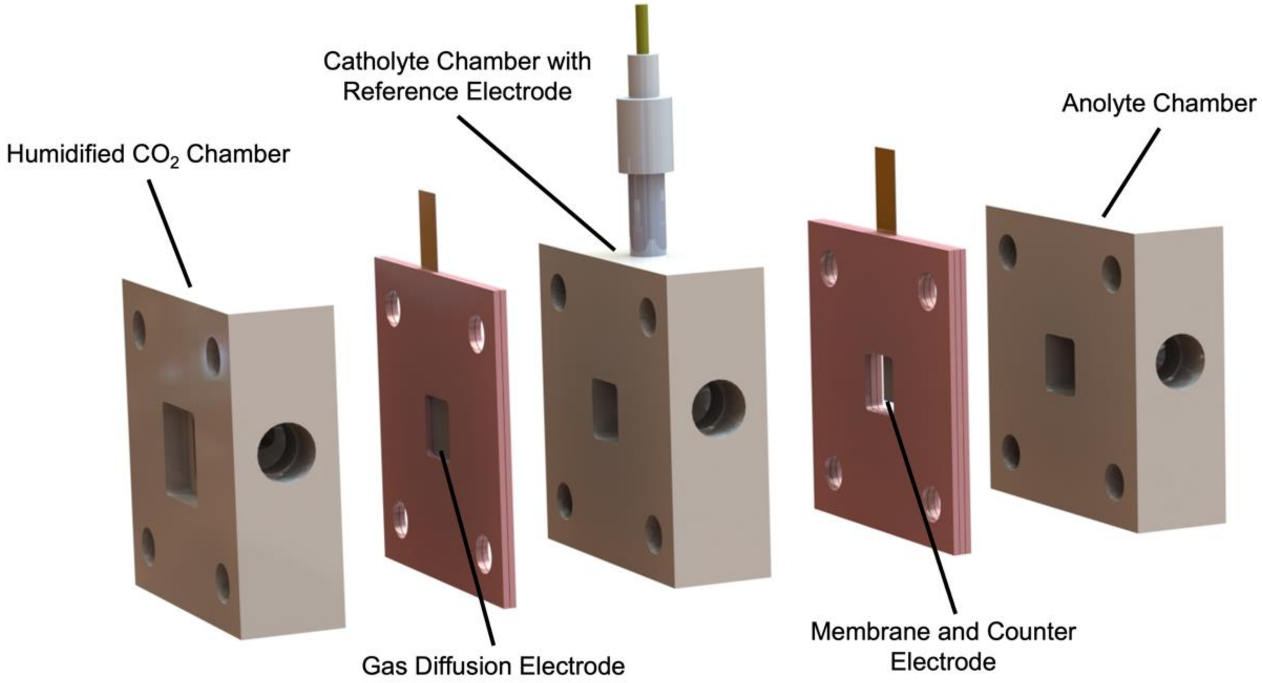

Figure S7. Schematics of the flow cell.

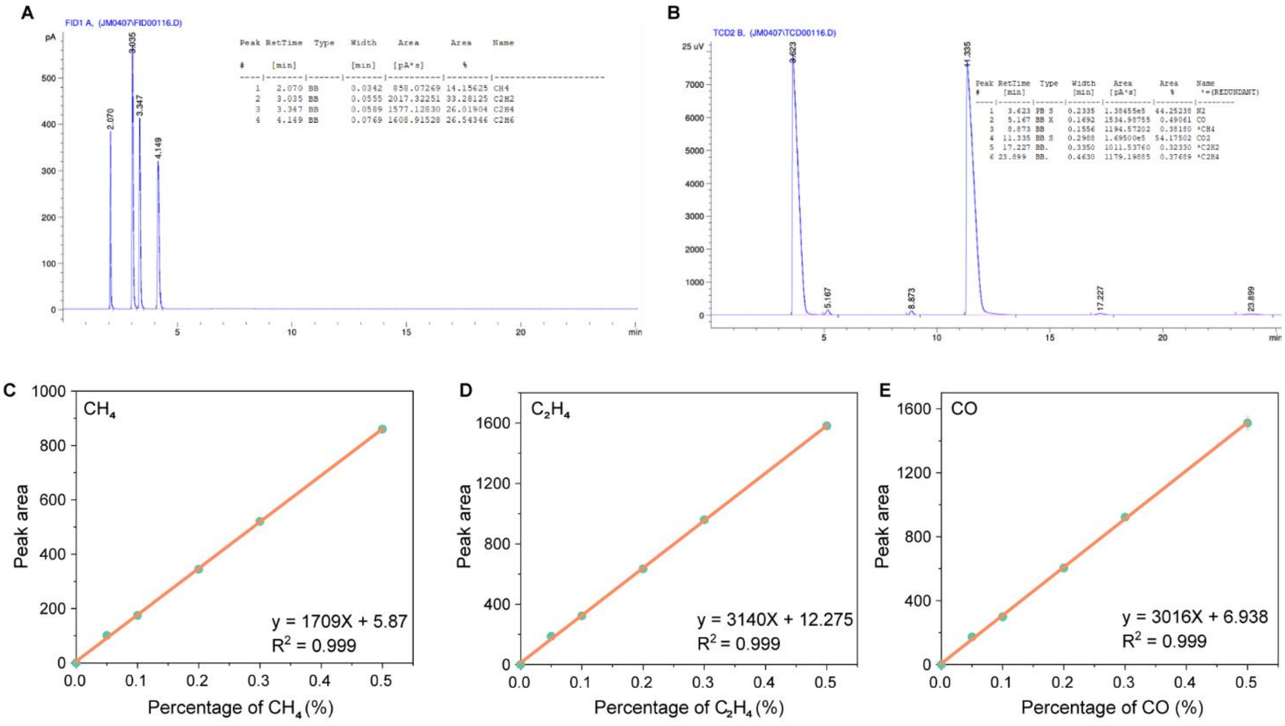

Figure S8. (A) Representative gas chromatography (GC) flame ionization detector (FID) spectrum for CO_2RR products; (B) Representative GC thermal conductivity detector (TCD) spectrum for CO_2RR products; (C-D) Standard curves of CH_4 (C), C_2H_4 (D), and CO (E). Source data are provided with this paper.

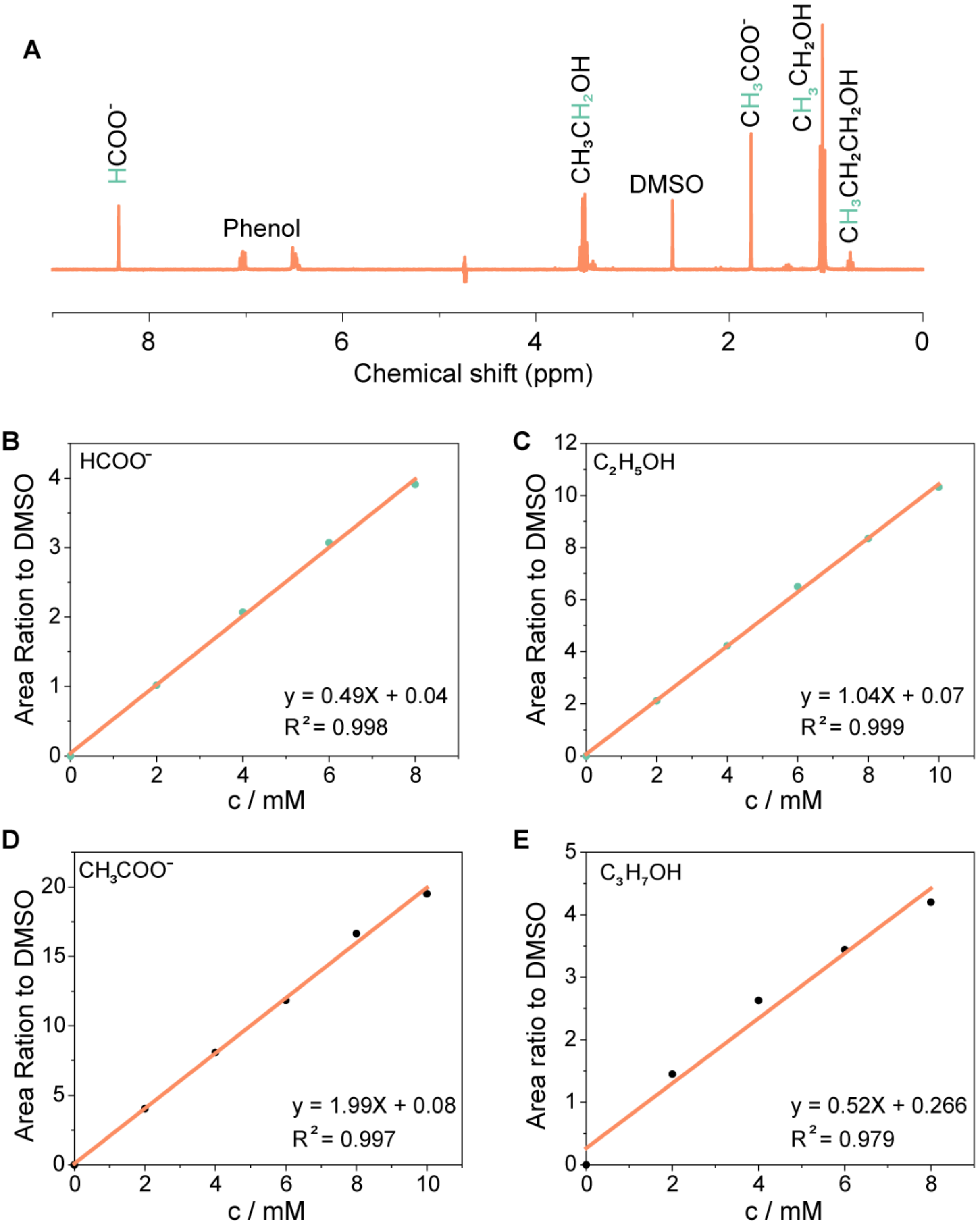

Figure S9. (A) Representative NMR spectrum for CO_2RR products; (B-E) Standard curves of $HCOO^-$ (B), C_2H_5OH (C), CH_3COO- (D) and C_3H_7OH (E). Source data are provided with this paper.

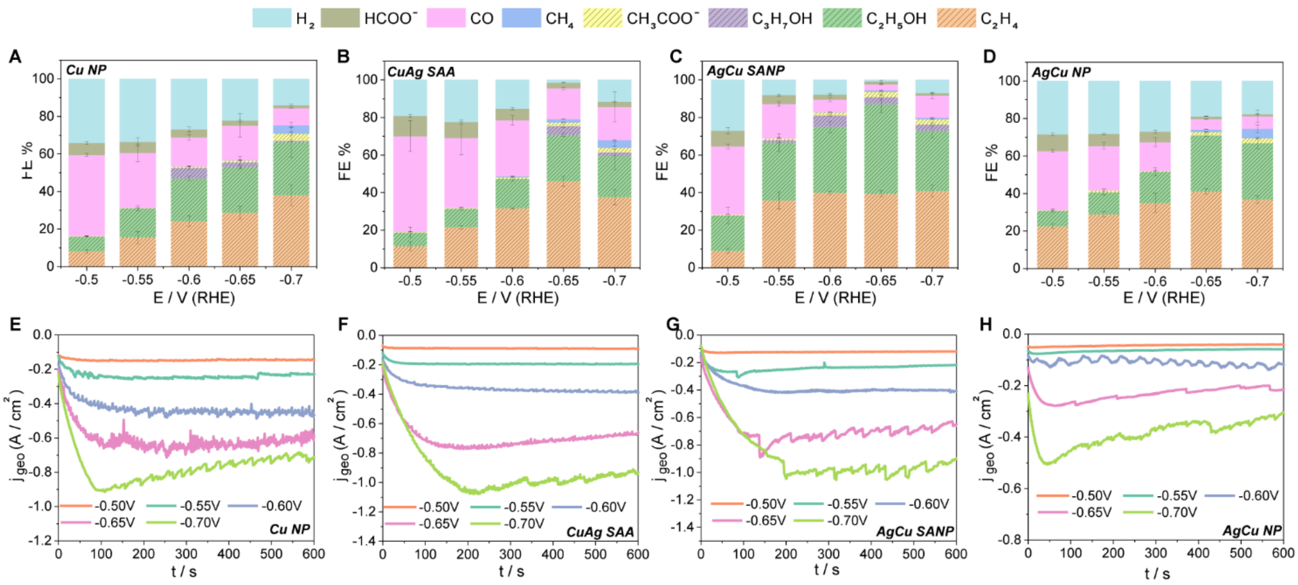

Figure S10. (A-D) Faradic efficiency (FE) results of Cu NP, AgCu SAA, AgCu SANP, and Ag NP under different potentials; (E-H) I-t curves of Cu NP, AgCu SAA, AgCu SANP, and Ag NP under different potentials. Noted that the FE of H_2 products from competitive hydrogen evolution reaction is not presented. All the error bars are obtained from three independent experiments. Source data are provided with this paper.

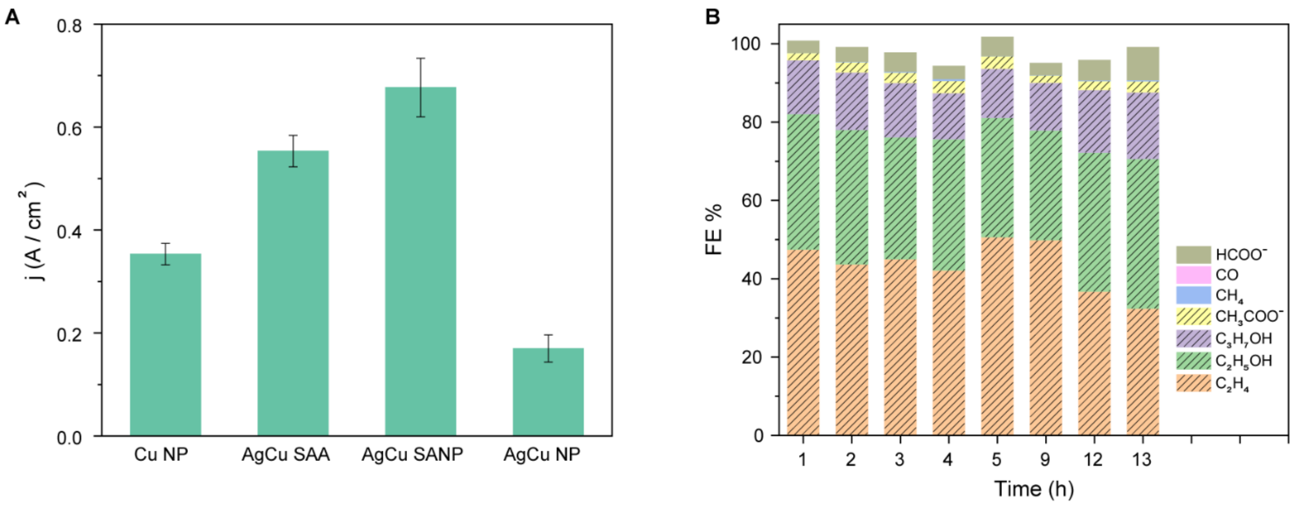

Figure S11. (A) The partial current density of C_{2+} products of Cu-based samples. All the error bars are obtained from three independent experiments; (B) The FE values of various products during the long-term stability test. Noted that the FE of H_2 products from competitive hydrogen evolution reaction is not presented. Source data are provided with this paper.

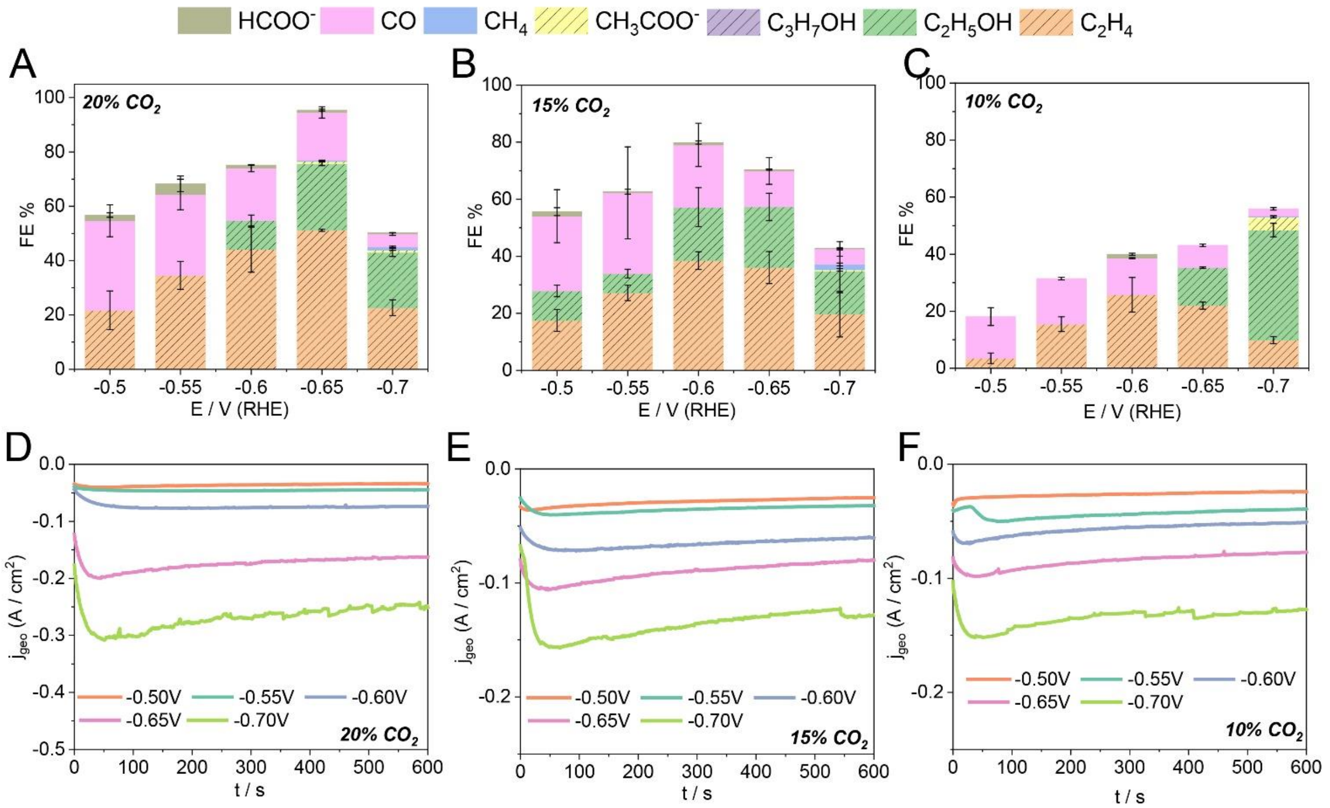

Figure S12. (A-C) FE results of AgCu SANP for CO_2RR feeding with different CO_2 concentrations, 20% (A), 15% (B) and 10% (C); (D-F) I-t curves of AgCu SANP for CO_2RR feeding with different CO_2 concentrations, 20% (D), 15% (E) and 10% (F). Noted that the FE of H_2 products from competitive hydrogen evolution reaction is not presented. All the error bars are obtained from three independent experiments. Source data are provided with this paper.

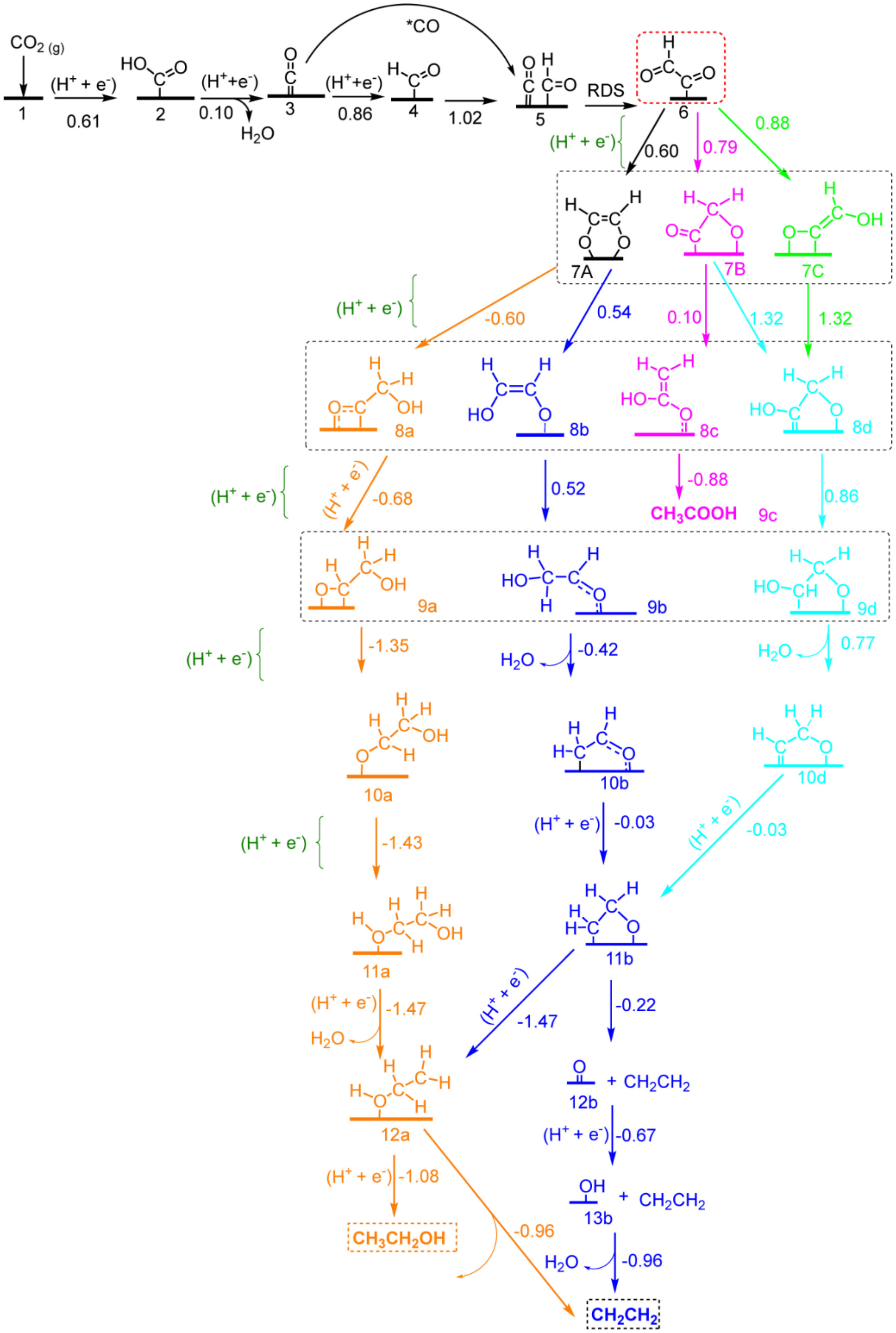

Figure S13. Proposed all mechanistic pathways for the formation of ethanol, ethylene, and acetic acid. The most less energetic pathways are given in the main text. The free energy of the reactions indicated in the arrows is in eV.

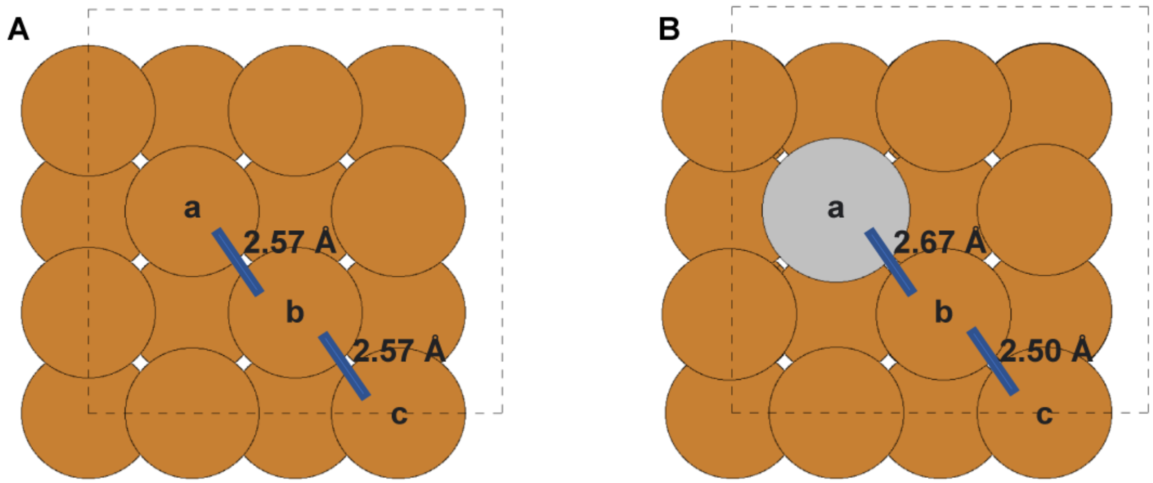

Figure S14. Cu-Cu bond distance comparison before Ag doping (A) and after Ag doping (B). Color code: Cu - brown, and Ag - light gray. Source data are provided with this paper.

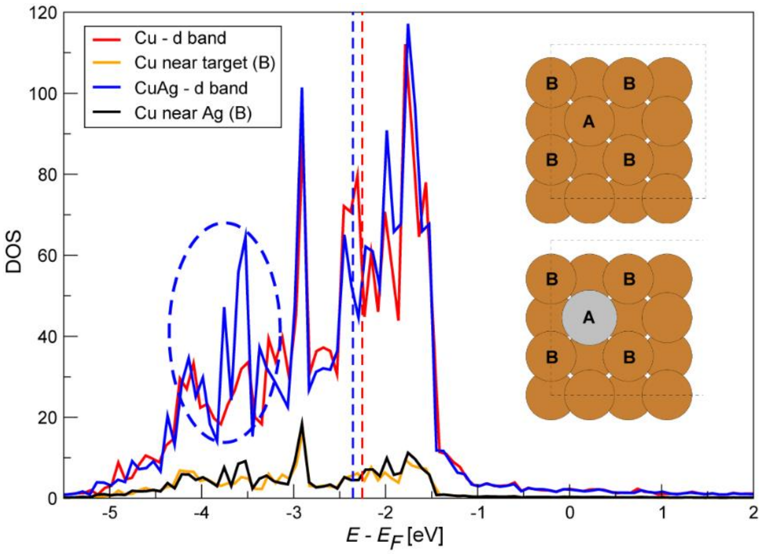

Figure S15. Projected *d*-band for pure Cu (red and orange lines) and Ag-doped Cu (blue and black lines) surfaces. Orange and black lines are the DOS projected on the *d*-band of the atoms near the target Cu (in pure Cu) and Ag atom (in CuAg). Insets: the target Cu and Ag atoms are indicated with the label 'A' whereas the first-neighbor Cu atoms are indicated with 'B'. The red and blue dashed lines indicate the *d*-band center of the pure Cu and CuAg surfaces, respectively. The Blue dashed oval indicates the excess DOS in CuAg. Color code: Cu - brown, and Ag - light gray.

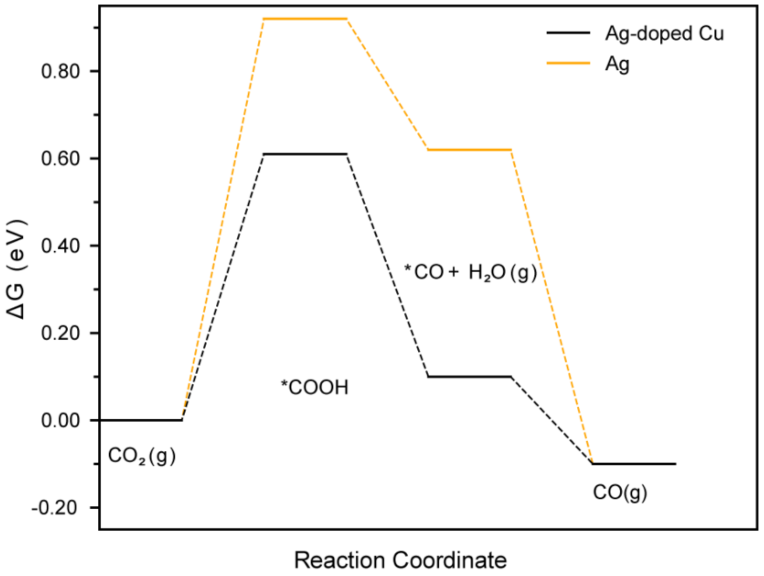

Figure S16. CO_2-to-CO reduction on Ag-doped Cu (black line) and Ag(100) (orange line).

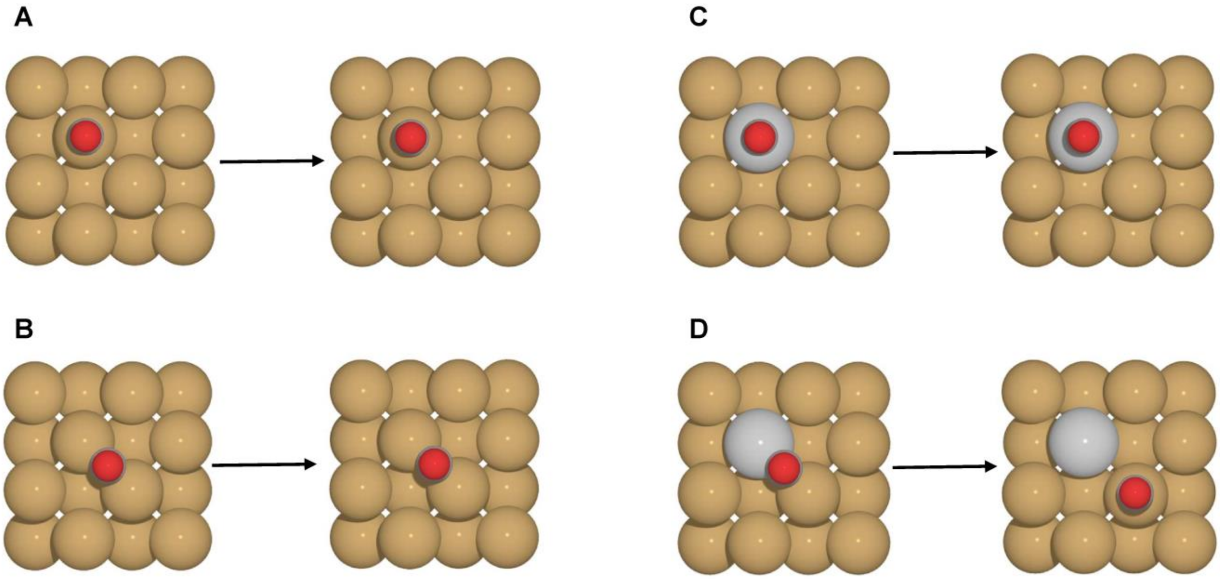

Figure S17. CO adsorbed structures before and after optimization for the ontop and bridge sites on pure Cu surface (A and B, respectively) and Ag doped-Cu surface (C and D, respectively). Color code: Cu - brown, Ag - light gray, C - dark gray, and O - red. Source data are provided with this paper.

Table S1 Fitted EXAFS parameters at the Cu and Ag K-edge for AgCu SANP, Ag foil, and Cu foil

AgCu SANP	CN	error	R(Å)	ERROR	E_0(eV)	σ^2(Å^2)	R-factor
Ag-Ag	7.32	0.73	2.859	0.009	2.4(7)	0.0080(1)	0.0103
Ag-Cu	1.03	0.09	**2.810**	0.048		0.0125(9)	
AgCu SANP	CN	error	R(Å)	ERROR	E_0(eV)	σ^2(Å^2)	R-factor
Cu-O	2.12	0.36	1.952	0.170	8(2)	0.0019(5)	0.0204
Cu-O	1.06	0.18	2.689	0.077		0.0030(2)	
Cu-Cu(oxide)	2.12	0.36	3.076	0.042		0.0118(1)	
Cu-Cu(metal)	1.42	0.65	2.548	0.018		0.0012(8)	
Cu-Ag	0.95	0.09	2.810	0.048		0.0008(5)	
Ag_foil	CN	error	R(Å)	ERROR	E_0(eV)	σ^2(Å^2)	R-factor
Ag-Ag	12		2.866	0.003	2.1(3)	0.0092(4)	0.0021
Cu_foil	CN	error	R(Å)	ERROR	E_0(eV)	σ^2(Å^2)	R-factor
Cu-Cu	12.00		2.544	0.003	4.5(5)	0.0090(4)	0.0011
Cu-Cu	6.00		3.595	0.022		0.0148(4)	
Cu-Cu-Cu	48.00		3.848	0.046		0.0202(5)	
Cu-Cu-Cu	48.00		4.373	0.032		0.0032(5)	
Cu-Cu	24.00		4.418	0.018		0.0125(2)	

CN: coordination number; R: distance; E_0: energy shift; σ^2: mean-square disorder; R-factor: goodness of EXAFS fitting). The numbers in the parentheses are the last digit error. Due to the complex mixture phases of Cu structures, Cu_2O, CuO, Cu atoms bonded to Ag in the Cu cluster, and Cu atoms not bonded to Ag in the Cu cluster, the coordination number of Cu-Ag extracted from Cu EXAFS spectral is just the demonstration of existence Cu-Ag.

Table S2 Performances comparison of AgCu SANP and reported results of alkaline solution in Figure 3D

Catalysts	NO.	j_{total} mA cm^{-2}	C_{2+} FE	Supplementary References
AgCu SANP	1	720	94	This work
$La_{2-x}CuO_{4-\delta}$	2	51.3	41.5	[1]
$Cu_xO_yC_z$	3	80	54	[2]
Cu-PzH	4	346	60	[3]
alkanethiol-modified Cu	5	255	64	[4]
CuO/Al_2CuO_4	6	600	70.1	[5]
F-Cu	7	400	70.4	[6]
Cu-CuI	8	700	71	[7]
$Cu_2P_2O_7$	9	350	73.6	[8]
Cu-SiO_x	10	300	75	[9]
electrodeposited Cu	11	150	75	[10]
Cu_3-Ag_3Au	12	230	77	[11]
B-Cu-Zn	13	200	79	[12]
Cu NPs with +1 valence	14	300	80	[13]
CuNi	15	250	80.5	[14]
Cu@Pb	16	400	81.6	[15]
Cu_3N_x	17	307	81.7	[16]
Cu Nanoribbons	18	347.9	82.3	[17]
Oxide-Derived Copper	19	341.5	83.8	[18]
SHKUST-1	20	400	88.4	[19]
$Cu(OH)_2$-derived Cu	21	217	87	[20]
N-C/Cu	22	300	93	[21]

Table S3 Gibbs free adsorption energies (ΔG_{ads}) of CO on Ag doped-Cu surface and pure Cu surface. CO adsorption on the bridge between Cu-Ag and 4-fold hollow sites is not stable (migrates to the nearby Cu sites).

Ag doped-Cu surface		Pure Cu surface	
Site type	ΔG_{ads} (eV)	Site type	ΔG_{ads} (eV)
Ontop of Cu	-0.60		
Ontop of Ag	-0.18	Ontop	-0.85
Bridge Cu-Cu	-0.63	Bridge	-0.87
Bridge Cu-Ag	unstable	Hollow (4-fold)	-0.90
Hollow (4-fold)	unstable		

Supplementary References

1. Zhu, J. *et al.* Cation-Deficiency-Dependent CO_2 Electroreduction over Copper-Based Ruddlesden–Popper Perovskite Oxides. *Angew. Chemie Int. Ed.* **61**, e202111670 (2022).
2. Sikdar, N. *et al.* A Metal–Organic Framework derived $Cu_xO_yC_z$ Catalyst for Electrochemical CO_2 Reduction and Impact of Local pH Change. *Angew. Chemie Int. Ed.* **60**, 23427–23434 (2021).
3. Wang, R. *et al.* Partial Coordination-Perturbed Bi-Copper Sites for Selective Electroreduction of CO_2 to Hydrocarbons. *Angew. Chemie Int. Ed.* **60**, 19829–19835 (2021).
4. Niu, Z. Z. *et al.* Hierarchical Copper with Inherent Hydrophobicity Mitigates Electrode Flooding for High-Rate CO_2 Electroreduction to Multicarbon Products. *J. Am. Chem. Soc.* **143**, 8011–8021 (2021).
5. Sultan, S., Lee, H., Yoon, A. & Choi, H. Interface rich CuO/Al_2CuO_4 surface for selective ethylene production from electrochemical CO_2 conversion Electrochemical Reduction of Carbon Dioxide View project. doi:10.1039/D1EE03861C.
6. Yan, X. *et al.* Boosting CO_2 electroreduction to C_{2+} products on fluorine-doped copper. *Green Chem.* **24**, 1989–1994 (2022).
7. Li, H. *et al.* High-Rate CO_2 Electroreduction to C_{2+} Products over a Copper-Copper Iodide Catalyst. *Angew. Chemie* **133**, 14450–14454 (2021).
8. Sang, J. *et al.* A Reconstructed $Cu_2P_2O_7$ Catalyst for Selective CO_2 Electroreduction to Multicarbon Products. *Angew. Chemie* **134**, e202114238 (2022).
9. Li, J. *et al.* Silica-copper catalyst interfaces enable carbon-carbon coupling towards ethylene electrosynthesis. *Nat. Commun. 2021 121* **12**, 1–10 (2021).
10. Zhang, X. *et al.* Selective and High Current CO_2 Electro-Reduction to Multicarbon Products in Near-Neutral KCl Electrolytes. *J. Am. Chem. Soc.* **143**, 3245–3255 (2021).
11. Xiong, L. *et al.* Breaking the Linear Scaling Relationship by Compositional and Structural Crafting of Ternary Cu–Au/Ag Nanoframes for Electrocatalytic Ethylene Production. *Angew. Chemie Int. Ed.* **60**, 2508–2518 (2021).
12. Song, Y. *et al.* B-Cu-Zn Gas Diffusion Electrodes for CO_2 Electroreduction to C_{2+} Products at High Current Densities. *Angew. Chemie Int. Ed.* **60**, 9135–9141 (2021).
13. Fan, Q. *et al.* Manipulating Cu Nanoparticle Surface Oxidation States Tunes Catalytic Selectivity toward CH_4 or C_{2+} Products in CO_2 Electroreduction. *Adv. Energy Mater.* **11**, 2101424 (2021).
14. Yan, Y. *et al.* Ultrathin CuNi Nanosheets for CO_2 Reduction and O_2 Reduction Reaction in Fuel Cells. *ACS Mater. Lett.* **3**, 1143–1150 (2021).

15. Wang, P. *et al.* Synergized Cu/Pb Core/Shell Electrocatalyst for High-Efficiency CO_2 Reduction to C_{2+} Liquids. *ACS Nano* **15**, 1039–1047 (2021).

16. Peng, C. *et al.* Lithiation-Enabled High-Density Nitrogen Vacancies Electrocatalyze CO_2 to C_2 Products. *Adv. Mater.* **33**, 2103150 (2021).

17. Huo, H., Wang, J., Fan, Q., Hu, Y. & Yang, J. Cu-MOFs Derived Porous Cu Nanoribbons with Strengthened Electric Field for Selective CO_2 Electroreduction to C_{2+} Fuels. *Adv. Energy Mater.* **11**, 2102447 (2021).

18. Chen, C. *et al.* The in situ study of surface species and structures of oxide-derived copper catalysts for electrochemical CO_2 reduction. *Chem. Sci.* **12**, 5938–5943 (2021).

19. Wen, C. F. *et al.* Highly Ethylene-Selective Electrocatalytic CO_2 Reduction Enabled by Isolated Cu−S Motifs in Metal–Organic Framework Based Precatalysts. *Angew. Chemie Int. Ed.* **61**, e202111700 (2022).

20. Zhong, D. *et al.* Coupling of Cu(100) and (110) Facets Promotes Carbon Dioxide Conversion to Hydrocarbons and Alcohols. *Angew. Chemie Int. Ed.* **60**, 4879–4885 (2021).

21. Wang, X. *et al.* Efficient electrically powered CO_2-to-ethanol via suppression of deoxygenation. *Nat. Energy 2020 56* **5**, 478–486 (2020).
